# Importance of prostate androgen-regulated mucin-like protein 1 in development of the bovine blastocyst

**DOI:** 10.1186/s12861-019-0195-7

**Published:** 2019-07-05

**Authors:** Adriana M. Zolini, Verónica M. Negrón-Pérez, Peter J. Hansen

**Affiliations:** 1 Department of Animal Sciences, D.H. Barron Reproductive and Perinatal Biology Research Program and Genetics Institute, University of Florida, PO Box 110910, Gainesville, Florida 32611-0910 USA; 2 Present address: Department of Animal and Poultry Sciences, Virginia Tech, Blacksburg, VA 24061 USA

**Keywords:** Embryo, Development, Blastocyst, PARM1, Gene expression, ER stress response

## Abstract

**Background:**

Prostate androgen-regulated mucin-like protein 1 (PARM1) is a pro-proliferative and anti-apoptotic glycoprotein involved in the endoplasmic reticulum (ER) stress response. A single nucleotide polymorphism in the coding region of *PARM1* has been associated with competence of bovine embryos to develop to the blastocyst stage. Here we tested the importance of PARM1 for development by evaluating consequences of reducing *PARM1* mRNA abundance on embryonic development and differentiation, gene expression and resistance to ER stress.

**Results:**

Knockdown of *PARM1* using an anti-*PARM1* GapmeR did not affect competence of embryos to develop into blastocysts but decreased the number of trophectoderm (TE) cells in the blastocyst and tended to increase the number of cells in the blastocyst inner cell mass (ICM). Treatment of embryos with anti-*PARM1* GapmeR affected expression of 4 and 3 of 90 genes evaluated at the compact-morula and blastocyst stage of development at days 5.5 and 7.5 after fertilization, respectively. In morulae, treatment increased expression of *DAB2, INADL,* and *STAT3* and decreased expression of *CCR2*. At the blastocyst stage, knockdown of *PARM1* increased expression of *PECAM* and *TEAD4* and decreased expression of *CCR7.* The potential role of PARM1 in ER stress response was determined by evaluating effects of knockdown of *PARM1* on development of embryos after exposure to heat shock or tunicamycin and on expression of *ATF6*, *DDIT3* and *EIF2AK3* at the compact morula and blastocyst stages. Both heat shock and tunicamycin reduced the percent of embryos becoming a blastocyst but response was unaffected by *PARM1* knockdown. Similarly, there was no effect of knockdown on steady-state amounts of *ATF6*, *DDIT3* or *EIF2AK3.*

**Conclusion:**

*PARM1* participates in formation of TE and ICM cells in early embryonic development but there is no evidence for the role of PARM1 in the ER stress response.

**Electronic supplementary material:**

The online version of this article (10.1186/s12861-019-0195-7) contains supplementary material, which is available to authorized users.

## Background

During cleavage stages of development, the mammalian embryo experiences a series of morphological, molecular, physiological and metabolic processes that culminate in the transformation of a single-cell, totipotent zygote into a multicellular blastocyst composed of differentiated trophectoderm (TE) and a pluripotent inner cell mass (ICM) [1]. Initially, blastomeres are transcriptionally silent and the embryo depends upon on maternally-derived mRNA stored in the oocyte for new protein synthesis. Epigenetic modifications and degradation of maternally-derived transcripts and proteins lead to activation of the embryonic genome at a species-specific cleavage stage [2]. In the bovine embryo, the first major round of genome activation is initiated at the 8–16 cell stage [3].

One of the genes expressed coincident with embryonic genome activation in the cow is prostate androgen-regulated mucin-like protein 1 (*PARM1)* [4] which encodes for a mucin-like type 1 transmembrane glycoprotein localized in the membrane of the endoplasmic reticulum (ER) [5, 6]. There was an association between a non-synonoymous single nucleotide polymoprhism (SNP) in the coding region of *PARM1* with competence of cultured bovine embryos to develop to the blastocyst stage [7]; embryos sired by heterozygous bulls were more likely to become a blastocyst than embryos sired by bulls homozygous for either SNP. Such a result is indicative that *PARM1* may play an important role in the preimplantation embryo.

There are at least two potential mechanisms by which PARM1 could affect development of the embryo to the blastocyst stage. The first is by regulation of cell differentiation because PARM1 can promote differentiation of cardiomyocytes [8] and adipocytes [9]. Actions on cardiomyocytes involve upregulation of expression of *BMP2* and *BMP4* [8]. Both of these bone morphogenetic proteins can regulate expression of transcription factor genes involved in differentiation [10]. Moreover, BMP4 promotes formation of trophoblastic cell lines from bovine blastocysts [11]. PARM1 could also improve embryonic development to the blastocyst stage by blocking ER stress induced apoptosis. Treatment of cardiomyocytes with silencing RNA targeting *PARM1* inhibited expression of *EITF2, AK3* and *ATF6* (participants in the unfolded protein response during ER stress) and increased apoptosis activated by ER stress inducers [5]*.* In addition, inhibition of ER stress improved the proportion of mouse and cattle embryos developing to the blastocyst stage [12, 13].

The objective of the present series of experiments was to determine whether *PARM1* plays an important role in development and differentiation of the blastocyst in the bovine preimplantation embryo. Experiments were conducted in vitro to test whether reducing transcript abundance for *PARM1* would affect the competence of the bovine embryo to develop to the blastocyst stage, alter the allocation of blastomeres in the blastocyst into TE and ICM lineages, change expression of genes important for embryonic development and alter embryo stress responses. Two types of stresses were evaluated. These were heat shock, which can reduce development to the blastocyst stage [14, 15] and which involves the ER stress response [16–18], and the glycosylation inhibitor tunicamycin, an ER stress inducer [19]. We also evaluated whether reducing transcript abundance for *PARM1* would alter expression of three genes involved in the ER stress response, namely *ATF6, DDIT3* and *EIF2AK3*. ATF6 and EIF2AK3 are ER sensory proteins which are kept inactivated by binding with ER-resident chaperones such as GRP78 and GRP94 [20]. Upon accumulation of misfolded proteins resulting from stress factors in the ER, the chaperones are occupied by the misfolded proteins, which results in the release and activation of the ER stress sensors, and subsequent activation of downstream signaling proteins such as CHOP. The *DDIT3* encoded protein, CHOP, acts as distal effector of ER stress response by mediating apoptotic signals [20].

## Results

### Embryo development and differentiation (experiment 1 and 2)

**Fig. 1:**
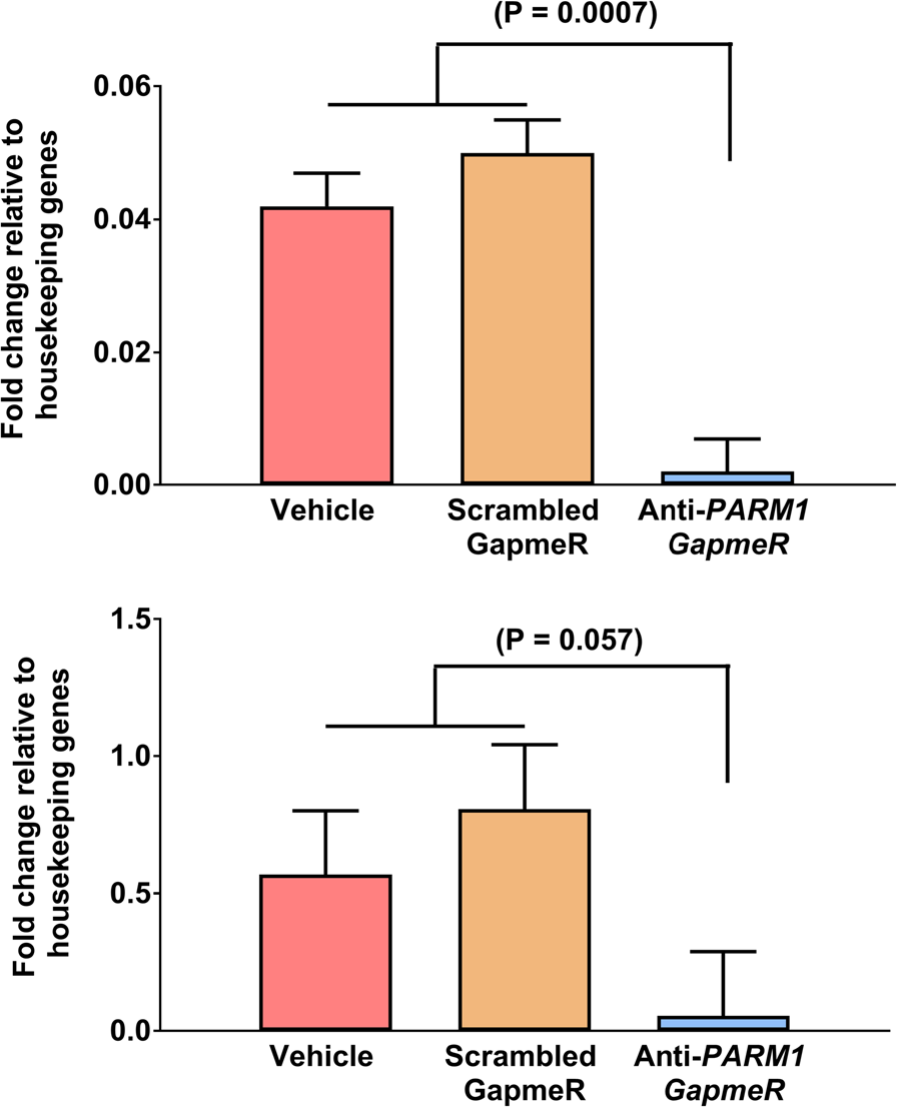
Effects of *PARM1* antisense oligonucleotide GapmeR on *PARM1* expression in embryos (Experiments 1 and 2). *Top*. Transcript abundance of *PARM1* in blastocysts harvested at day 7.5 after fertilization (Experiment 1*). Bottom*. Transcript abundance of *PARM1* in compact morulae harvested at day 5.5 after fertilization (Experiment 2). Data are least-squares means ± SEM of fold change relative to housekeeping genes. The *P*-values for effects of treatment as determined by orthogonal contrasts are indicated by the value above the bars

**Fig. 2:**
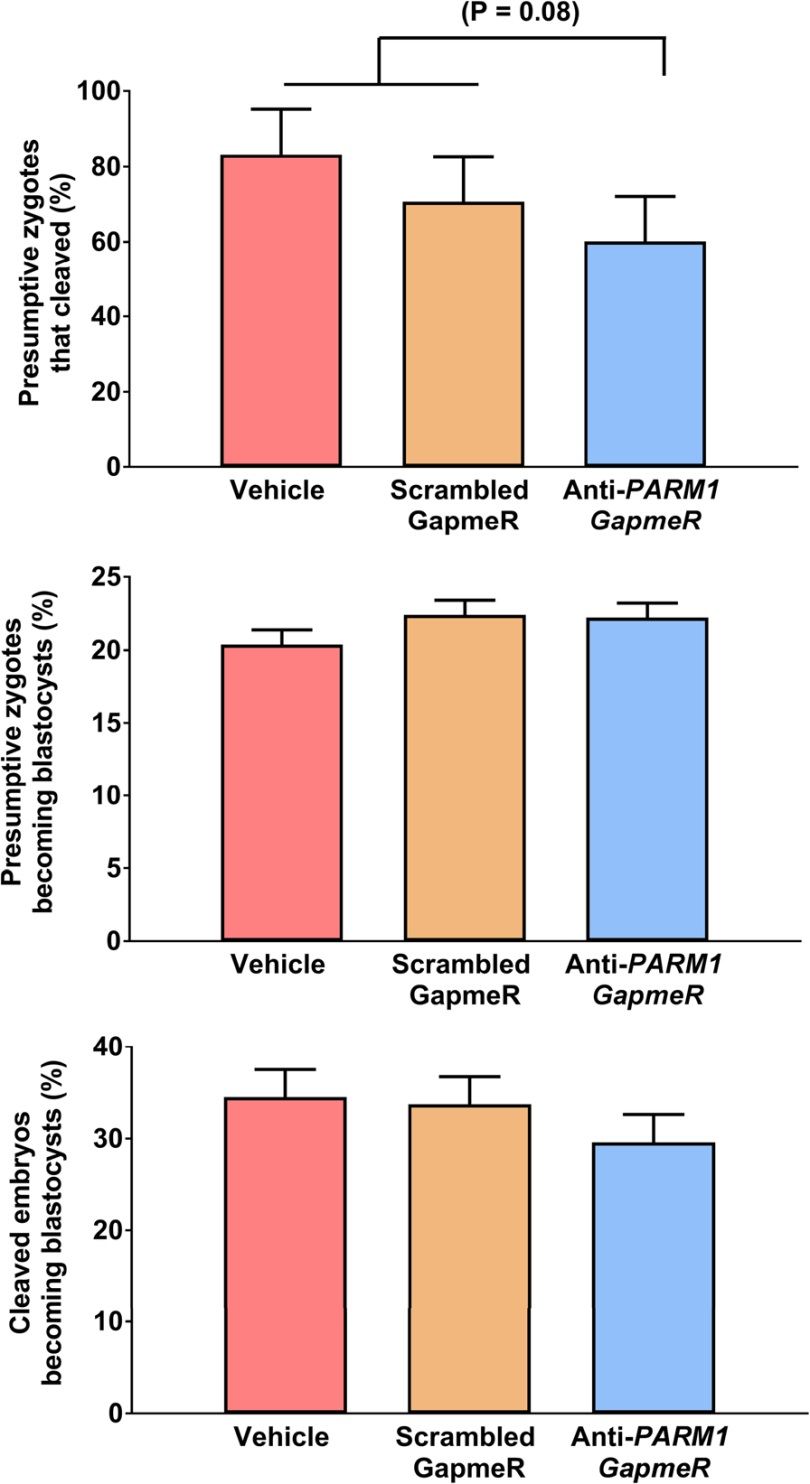
Effects of *PARM1* antisense oligonucleotide GapmeR on competence of embryos to become a blastocyst at day 7.5 after fertilization (Experiment 1). Data are least-squares means ± SEM. *Top***.** Percent of presumptive zygotes that cleaved at day 3.5 after fertilization. *Middle***.** Percent of presumptive zygotes that became a blastocyst at day 7.5 after fertilization. *C.* Percent of cleaved embryos that became a blastocyst at day 7.5 after fertilization. The P-values for effects of treatment as determined by orthogonal contrasts are indicated by the value above the bars

**Fig. 3:**
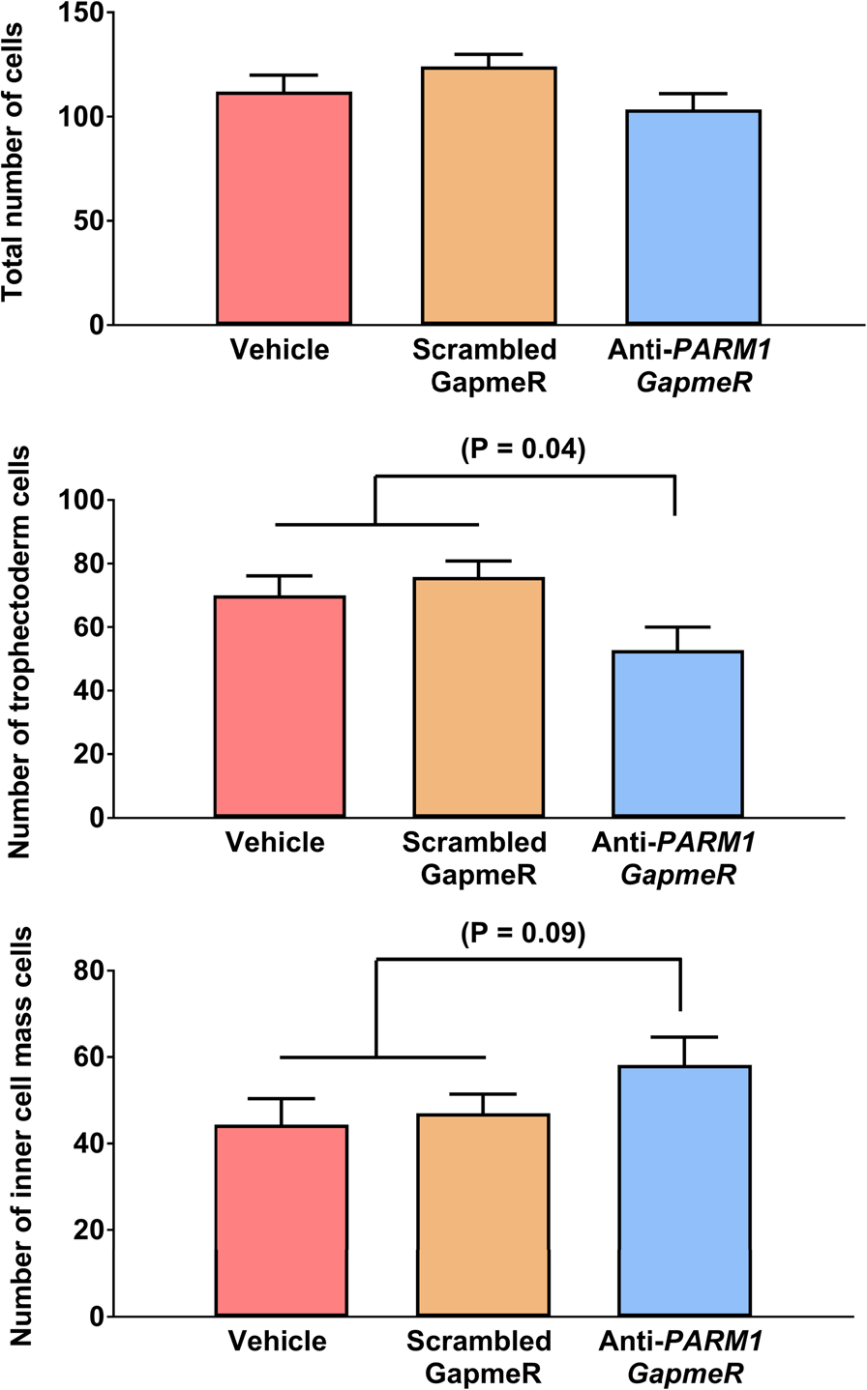
Effects of a *PARM1* antisense oligonucleotide GapmeR on number of total cells (top panel), trophectoderm cells (middle panel) and inner cell mass cells (bottom panel) in blastocysts (Experiment 1). Data are least-squares means ± SEM. The P-values for effects of treatment as determined by orthogonal contrasts are indicated by the value above the bars

The effectiveness of anti-PARM1 GapmeR for reducing abundance of *PARM1* in morula and blastocyst stage embryos was confirmed using qPCR (Fig. 1). Treatment with anti-*PARM1* GapmeR tended to decrease (*P* = 0.08) the percent of presumptive zygotes that cleaved at day 3.5 after fertilization (Fig. 2 top panel) but had no effect on the percent of either presumptive zygotes or cleaved embryos developing to the blastocyst stage (Fig. 2 middle panel and Fig. 2 bottom panel). Knockdown of *PARM1* did not affect total number cells in blastocysts (Fig. 3 top panel) but decreased (*P* = 0.04) the number of trophectoderm cells (Fig. 3 middle panel) and tended to increase (*P* = 0.09) the number of ICM cells (Fig. 3 top panel).

### Transcript abundance for genes related to early embryonic development (experiments 1 and 2)

Effects of reducing transcript abundance of *PARM1* on gene expression was evaluated by determining steady-state amounts of 90 genes selected for their role in embryonic development including those identified as markers of epiblast, TE or hypoblast, cell signaling pathways, genes involved in epigenetic modification and genes involved in tight junctions and, cell polarity. Gene expression was evaluated at morula (Experiment 2) and blastocyst stages (Experiment 1).

**Fig. 4:**
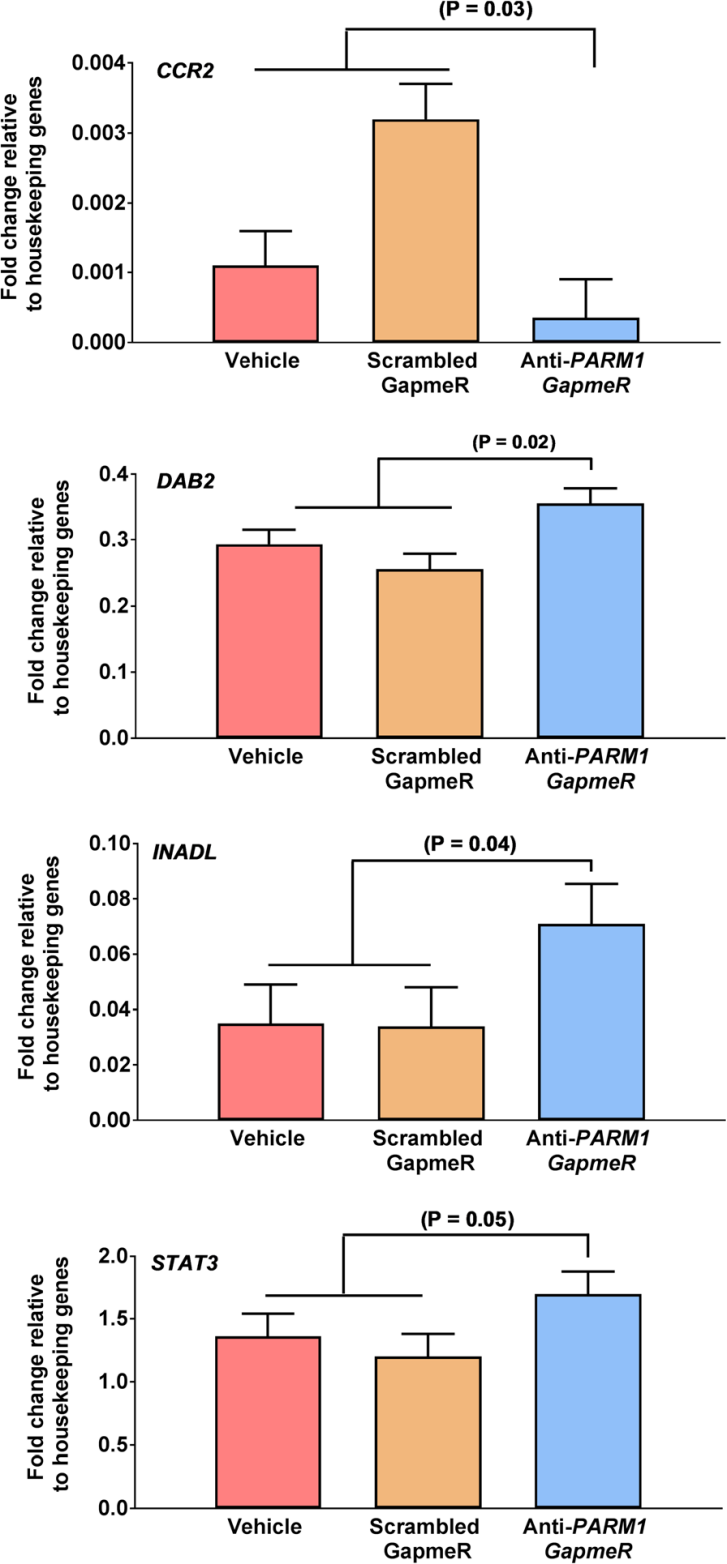
Effects of *PARM1* antisense oligonucleotide GapmeR on transcript abundance in morulae at day 5.5 after fertilization as determined by high-throughput quantitative PCR (Experiment 2). Data are represented as fold change relative to geometric means of housekeeping genes and are least-squares means ± SEM. The P-values for effects of treatment as determined by orthogonal contrasts are indicated by the value above the bars

**Fig. 5:**
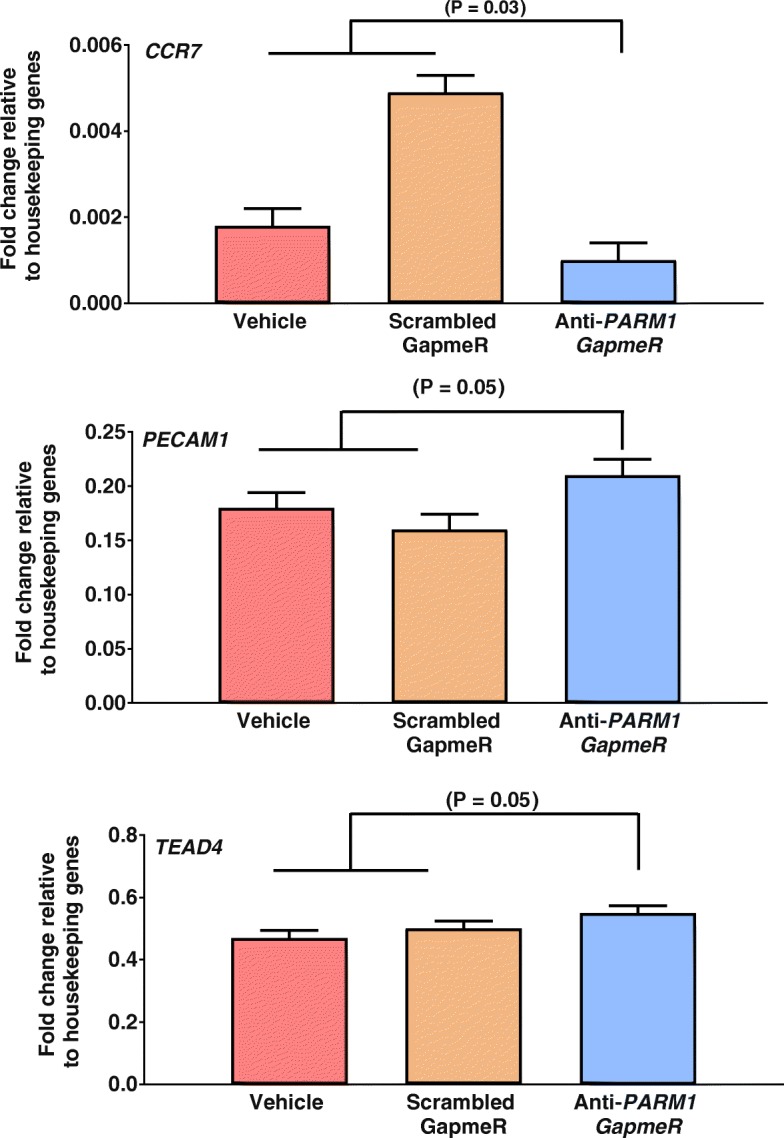
Effects of *PARM1* antisense oligonucleotide GapmeR on transcript abundance in blastocysts at day 7.5 after fertilization as determined by high-throughput quantitative PCR (Experiment 1). Data are represented as fold change relative to geometric means of housekeeping genes and are least-squares means ± SEM. The P-values for effects of treatment as determined by orthogonal contrasts are indicated by the value above the bars

Least-squares means of fold change relative to housekeeping for all genes analyzed are shown in Additional file 1. At the morula stage (Day 5.5), treatment of embryos with anti-*PARM1* GapmeR increased expression of *DAB2* (*P* = 0.02), *INADL* (*P* = 0.04), and *STAT3* (*P* = 0.05) and decreased (*P* = 0.03) amounts of mRNA for *CCR2* (Fig. 4). At the blastocyst stage, treatment of embryos with anti-*PARM1* GapmeR increased expression of *PECAM1* (P = 0.05), *TEAD4* (P = 0.05), and decreased expression of *CCR7* (P = 0.03*)* (Fig. 5)*.*

### Effect of knockdown of *PARM1* on development of embryos after heat shock (experiment 3)

**Fig. 6:**
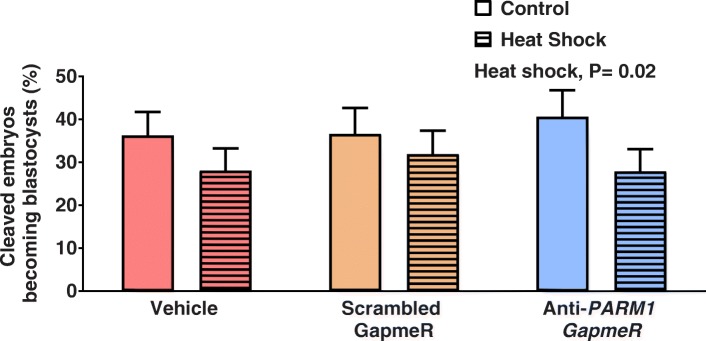
Effects of *PARM1* antisense oligonucleotide GapmeR and heat shock at day 5.5 after fertilization on competence of cleaved embryos to become a blastocyst at day 7.5 after fertilization (Experiment 3). At day 5.5 after fertilization, embryos were subject to a heat shock of 41 °C for 24 h (Heat Shock; hatched bars) or were continued to be cultured at 38.5 °C (i.e., Control; solid bars). All embryos were cultured at 38.5 °C thereafter. Data are least-squares means ± SEM. Heat shock treatment decreased (*P* = 0.02) the percent of cleaved embryos becoming a blastocyst at day 7.5 after fertilization. There was no no effect of GapmeR treatment or interaction between effects of heat shock and GapmeR treatment on percent of cleaved embryos becoming a blastocyst at day 7.5 after fertilization

Heat shock treatment decreased (P = 0.02) the percent of cleaved embryos that became a blastocyst at Day 7.5 but there was no effect of anti-*PARM1* GapmeR or the interaction between anti-*PARM1* GapmeR and heat shock (Fig. 6).

### Effect of knockdown of *PARM1* on development of embryos after tunicamycin treatment (experiment 4)

**Fig. 7:**
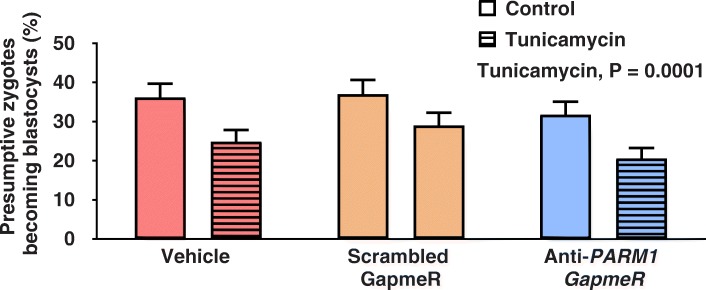
Effects of *PARM1* antisense oligonucleotide GapmeR and tunicamycin at day 5.5 after fertilization on competence of embryos to become a blastocyst at day 7.5 after fertilization (Experiment 4). Data are least-squares means ± SEM. At day 5.5 after fertilization, 2.5 μL of medium in the culture drop was replaced with 2.5 μL SOF-BE2 containing vehicle (Control; solid bars) or tunicamycin (0.5 μg/mL tunicamicyn so that the final concentration would be 0.05 μg/mL; hatched bars). Treatment with tunicamycin decreased (*P* = 0.0001) the percent of presumptive zygotes becoming a blastocyst at day 7.5 after fertilization. There was no effect of GapmeR treatment or interaction between effects of tunicamycin treatment and GapmeR treatment on blastocyst development

Tunicamycin decreased (*P* = 0.0001) the percent of presumptive zygotes that became a blastocyst at day 7.5 but there was no effect of anti-*PARM1* GapmeR or the interaction between anti-*PARM1* GapmeR and tunicamycin (Fig. 7).

### Expression of genes involved in ER stress (experiments 5 and 6)

**Fig. 8:**
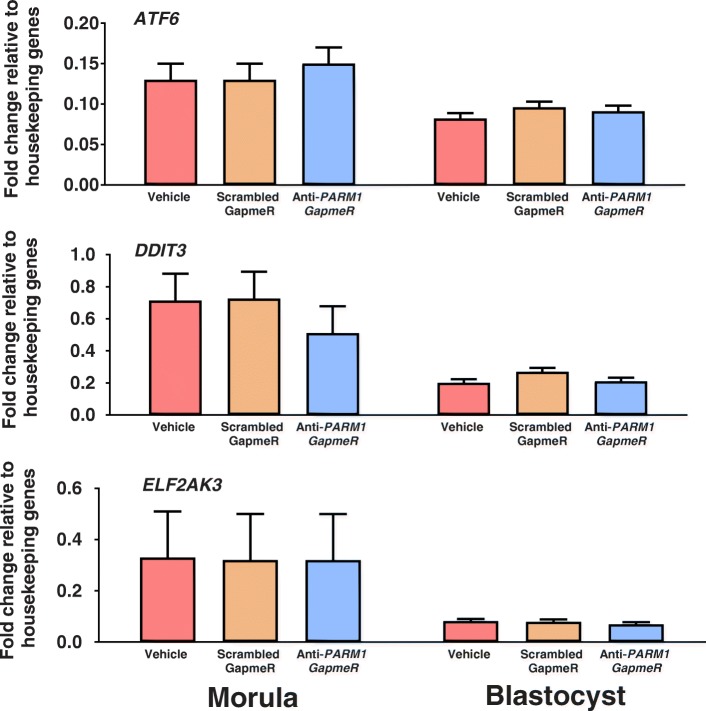
Effects of *PARM1* antisense oligonucleotide GapmeR on transcript abundance for selected genes involved in endoplasmic reticulum stress response in morulae (Experiment 5) and blastocysts (Experiment 6). Data are least-squares means ± SEM. There was no treatment effect on gene expression of *ATF6*, *DDIT3* and *ELF2AK3* for morula-stage embryos collected at day 5.5 after fertilization and for blastocyst-stage embryos collected at day 7.5 after fertilization

Treatment with anti-*PARM1* GapmeR had no effect on steady-stage amounts of mRNA for *ATF6*, *DDIT3* or *EIF2AK3* at either the compact morula or blastocyst stages of development (Fig. 8).

## Discussion

Results reported here show that *PARM1* is not required for development of the embryo to the blastocyst stage, does not play a role in determining whether an embryo can develop to the blastocyst stage after two treatments that can induce the ER stress response, heat shock [16–18] or tunicamycin exposure [19], and is not determinative for expression of key genes involved in the ER stress response. Nonetheless, PARM1 does play a role in the process of differentiation of the embryo into TE and ICM cells at the blastocyst stage. This is so because reducing *PARM1* transcript abundance decreased the number of TE cells and tended to increase the number of ICM cells.

PARM1 is a pro-proliferative and anti-apoptotic glycoprotein [6, 21] and it is possible that *PARM1* is involved in embryonic differentiation by promoting TE cell proliferation or reducing apoptosis of TE cells. If so, knockdown of *PARM1* could result in lower TE cell number because of reduced proliferation or increased apoptosis. However, such a putative mechanism does not explain why knockdown of *PARM1* also increased ICM cell number. A more likely explanation for the change in numbers of TE and ICM cells caused by *PARM1* knockdown is that PARM1 is involved in allocation of blastomeres towards the TE or ICM lineages.

Examination of the effects of *PARM1* knockdown on gene expression in embryos at the compact morula or blastocyst stage did not provide a clear picture of the mechanism by which PARM1 could favor allocation of blastomeres to TE rather than ICM. Most genes were not affected by treatment and many of the genes significantly affected were either very lowly expressed (*CCR2* for compact morulae and *CCR7* for blastocysts) or the magnitude of the effect was small. Three genes implicated in differentiation of the bovine blastocyst were not affected by anti-*PARM1* GapmeR (see summary of results in Additional file 1). These were *CDX2* [22–24] and *YAP1* [25], important for TE formation, and *POU5F1* [26] required for epiblast formation. There was also no effect of *PARM1* knockdown on expression of *BMP4* even though overexpression of *PARM1* in cardiomyocytes causes upregulation of *BMP2* and *BMP4* expression [8]. Expression of another gene important for TE differentiation in cattle, *TEAD4* [24, 25, 27]*,* was slightly increased in blastocysts produced in the presence of anti-*PARM1* GapmeR. TEAD4 is required for expression of *CDX2* [24] so an increase in TEAD4 caused by the *PARM1* knockdown is inconsistent with the idea that PARM1 is affecting TE cell numbers by regulation of *TEAD4* expression.

Two genes whose expression was increased by PARM1, namely *INADL* at the compact morula stage and *PECAM1* at the blastocyst stage, are involved in junctional complexes between cells. *INADL* encodes for a protein involved in tight junction formation [28] which is exclusive to TE cells and is essential for blastocoele formation in the mouse [29]. *PECAM1* encodes for a transmembrane cell-adhesion protein involved in formation of adherens junctions [30]. *PARM1* is a mucin present not only in the ER but also in the plasma membrane [31]. Perhaps PARM1 participates in cell-cell contact and reduction in amounts of PARM1 upon GapmeR treatment results in upregulation of these two genes involved in formation of junctional complexes. *PARM1* knockdown also increased expression of *DAB2* in compact morulae. *DAB2* encodes a protein important for endocytosis [32] and PARM1 can be localized to early endosomes [31]. It has been proposed that endocytosis is important for cell polarization and tight junction formation in the mouse embryo [33] and perhaps actions of *PARM1* to promote TE differentiation involve actions on endosome formation.

The knockdown of *PARM1* caused increased expression of *STAT3* at the compact-morula stage. In the bovine embryo, inhibition of *STAT3* causes reduced ICM cell numbers and expression of *NANOG* [34] so the increase in expression of *STAT3* caused by *PARM1* knockdown could conceivably contribute to the increased numbers of ICM cells in blastocysts cultured with anti-*PARM1* GapmeR. STAT3 participates in ICM maintenance in the mouse by upregulating pluripotency genes *POU5F1* and *NANOG* [35].

Implications of a change in the relative numbers of TE and ICM cells in the blastocyst for subsequent competence to establish and maintain pregnancy are not known. The ratio of number of TE cells to number of ICM cells can be affected by culture conditions for in vitro produced embryos [36]. Loss of the embryonic disk at day 14–15 is a frequent occurrence for embryos produced in vitro [37, 38]. In vitro, morulae that were classified as poor based on morphological criteria had fewer ICM cells when they became hatched blastocysts [39]. Perhaps, overactivation of *PARM1* expression for the embryo produced in vitro can affect the ratio of TE:ICM .

PARM1 is also present in the ER where it plays a role in the ER stress response [5]. There is no evidence, however, that PARM1 is involved in modulating the ER stress response in the bovine embryo. Knockdown of *PARM1* did not make embryos more sensitive to the anti-developmental effects of heat shock or tunicamycin. Heat shock can activate the ER stress response in cultured cells [16, 17] and survival of cardiomyocytes to this insult was enhanced when the ER stress response was attenuated [18]. Lack of effect of *PARM1* knockdown on embryonic response to heat shock is indicative that either *PARM1* is not involved in the ER stress response in the bovine embryo or the ER stress response was not activated by heat shock. To verify that *PARM1* is not involved in ER stress response during early embryonic development embryos were treated with tunicamycin at day 5.5 after fertilization. As reported previously [40], tunicamycin induces ER stress response by blocking N-glycosylation of proteins and can impair compaction and blastocyst formation. In the present experiment, tunicamycin had a large negative effect on competence of the embryo to become a blastocyst but this effect was not modified by anti-*PARM1* GapmeR treatment. This result is also inconsistent with a role for PARM1 in the ER stress response. Further evidence against the involvement of PARM1 in the ER stress response in the bovine embryo was the observation that knockdown of *PARM1* had no effect in the expression of three genes involved in the ER stress response, *ATF6, DDIT3,* and *EIF2AK3*.

The fact that treatment with anti-*PARM1* GapmeR did not affect blastocyst formation implies that the SNP in *PARM1* that was earlier associated with blastocyst development [7] is not a causative mutation. This SNP is a G → C missense mutation that induces an amino acid change from glycine to alanine at position 232. One possibility is that the SNP is in linkage disequilibrium with the actual causative mutation. The discovery of the relationship between the *PARM1* SNP and embryonic development [7] was based on the effect of *PARM1* genotype of the sire used to produce embryos and subsequent percent of embryos that became blastocysts in culture. It is also possible, therefore, that the SNP affects sperm function. Recent results also failed to find a relationship between the *PARM1* SNP and fertility in lactating dairy cows [41].

## Conclusion

Present results indicate that *PARM1* should be considered as part of the gene network involved in TE and ICM formation. As such, it is the first gene encoding for an ER protein found to be implicated in cellular differentiation of the blastocyst. Future studies should be done to investigate the precise location of PARM1 in embryonic cells and the proteins that interact with PARM1 to affect differentiation of the morula into TE and ICM.

## Methods

### In vitro production of bovine embryos

All experiments were performed with embryos produced in vitro using a previously-described protocol [4, 25, 42, 43]. Briefly, ovaries from *Bos taurus* or admixtures of *B*. *taurus* and *B*. *indicus* cattle were used to harvest cumulus-oocyte complexes (COC). Harvest was accomplished by by slicing visible ovarian follicles (2–8 mm in diameter) and rinsing the sliced ovary in oocyte washing medium (MOFA Global, Verona, WI, USA). The COC were matured for 22 h at 38.5 °C in a humidified atmosphere of 5% (v/v) CO_2_ in groups of 10 in 50 μL oil-covered drops of a commercial oocyte maturation medium (BO-IVM Biosciences, Cornwall, United Kingdom). Fertilization of matured oocytes was performed by placing up to 300 COC with frozen-thawed, Isolate® (Irvine Scientific, Santa Ana, CA, USA)-purified sperm (1 × 10^6^/ml) pooled from three bulls of various breeds. A replicate was considered all oocytes collected on one day and different pools of sperm were used for different replicates. Fertilization took place in n-vitro fertilization-Tyrode’s albumin lactate pyruvate (IVF-TALP) that contained 20 μM penicillamine, 10 μM hypotaurine and 0.007 μM epinephrine. Fertilization proceeded for 12–14 h at 38.5 °C in a humidified gas atmosphere of 5% (v/v) CO_2_, and with the balance nitrogen. Cumulus cells were removed from presumptive zygotes after fertilization by vortexing with 1000 U/ml hyaluronidase in ~ 0.5 ml HEPES-TALP. After washing 3x in HEPES-TALP, groups of 15 presumptive zygotes were cultured at 38.5 °C in a humidified gas atmosphere of 5% (v/v) CO_2_, 5% (v/v) O_2_ with the balance nitrogen in 25 μl oil-covered drops of synthetic oviductal fluid-bovine embryo 2 (SOF-BE2).

### Knockdown of *PARM1*

Knockdown was performed using GapmeR LNA™ antisense oligonucleotides from Exiqon (Woburn, MA, USA) using procedures employed previously to knockdown mRNA for *WBP1* [4], *YAP1* [25] and *AMOT* [25]. Two GapmeR were designed by Exiqon: one to target the *PARM1* sequence and a scrambled version of the same sequence used as a negative control. The sequence that targeted *PARM1* (termed anti-*PARM1*) was 5′-GTGAAGTAGGTGTGAG-3′. The sequence for the scrambled negative control was 5′-TTGGATGGTGGAGAGA-3′. Sequences were evaluated to ensure that they do not hybridize to other genes. Presumptive zygotes (i.e., oocytes exposed to sperm and placed in embryo culture) were randomly treated at 22 h post insemination with either 5 μM anti-*PARM1* GapmeR, 5 μM scrambled GapmeR, or vehicle (RNA free water) added to SOF-BE2 culture medium. A single administration of treatment was applied and embryos were cultured with the relevant treatment for the entire duration of culture without addition of additional GapmeR.

### Determination of the number of ICM and TE cells by immunofluorescence

Cells that were TE were distinguished from cells in the ICM based on nuclear labeling for CDX2 using procedures similar to those previously published [4, 25, 44, 45]. The primary antibody was ready-to-use mouse monoclonal antibody against CDX2 (BioGenex, Fremont, CA, USA; reference AM392-5 M, lot AM3920917) For randomly-selected embryos, mouse 1gG (1 μg/mL) was used as a negative control. The second antibody was 1 μg/ml fluorescein isothiocyanate labeled goat anti-mouse IgG. Nuclei were labeled with 1 μg/ml Hoechst 33342. Fluorescence was observed with a Zeiss Axioplan 2 epifluorescence microscope (Zeiss, Gottingen, Germany) with a 40x objective and using Zeiss filter sets 02 (blue) and 03 (green). Digital images were acquired using AxioVision software (Zeiss) and a high-resolution black and white Zeiss AxioCam MRm digital camera. Total cell number was determined by counting nuclei labeled with Hoescht 33,342, number of TE cells was determined by counting nuclei positive for CDX2 and number of ICM cells was determined by subtracting number of TE cells from total cell number.

### RNA isolation

Pools of embryos were treated with 0.1% (w/v) protease from *Streptococcus griseus* in DPBS containing 1% (w/v) polyvinylpyrrolidone to remove the zona pellucida, washed three times in 50 μl droplets of DPBS containing 1% PVP, placed in 5 mL DEPC-treated DPBS-1% PVP in microcentrifuge tubes, snap-frozen in liquid nitrogen and stored at − 80 °C until further analysis. For RNA extraction, the PicoPure RNA isolation kit (Applied Biosystems, Carlsbad, CA, USA) was used following the manufacturer’s instructions by loading onto MiraCol purification column and eluting with buffer. Isolated RNA (10 μl) was treated with 1 μL (2 U) of DNaseI (New England Biolabs, Ipswich, MA, USA) for removal of DNA contamination prior to reverse transcription.

### Analysis of expression of individual genes using quantitative PCR

Reverse transcription was performed with the High Capacity cDNA Reverse Transcription Kit (Applied Biosystems). A negative control without reverse transcriptase was included for each stamples. The PCR was carried out using a CFX96 Real-Time PCR detection System (Bio-Rad, Hercules, CA, USA) and the SsoFast EvaGreen Supermix® with Low ROX (Bio-Rad) as described elsewhere [4]. Genes whose expression was determerined were *ATF6, DIT3, ELF2AK3*, and *PARM1.* Primer sequences are detailed in Table 1. Validation for each set of primers included a standard curve with a slope between − 3 and − 3.3, verification that melting curves incdicated a single, specific product, confirmation of amplicon size by agarose gel electrophoresis and Sanger sequencing of the PCR product to condirm sequence identify.

**Table 1:** Nucleotide sequence of the forward and reverse primers used for quantitative PCR

Gene	Accession number	Forward primer	Reverse primer
*ATF6*	BC120388	TCATAGGCACGAAGTGGAAAG	CCAGAGCACCCTGAAGAATAC
*DDIT3*	NM_001078163	CTCTCTGGCTTGGCTTACTG	GTTCTTCCTTGGTCTTCCTCTT
*EIF2AK3*	NM_001098086.1	GCTATCAAGAGAATCCGTCTCC	TCTCACAATTCCTGGGTGTTC
*PARM1*	NM_001075771.2	AGCTCTACTCTCACACCTACTT	GTGGTGGTCTCCATGTCTATC
*GAPDH*	NM_001034034	TTCAACGGCACAGTCAAGG	ACATACTCAGCACCAGCATCAC
*YWHAZ*	BM446307	GCATCCCACAGACTATTTCC	GCAAAGACAATGACAGACCA

### Analysis of expression of individual genes using high-throughput quantitative PCR

The Fluidigm qPCR microfluidic device Biomark HD system was used to analyze the effect of *PARM1* knockdown on 96 selected genes using primer pairs and protocol published earlier [25]. Primers were designed by Fluidigm Delta Gene assays (Fluidigm Co., San Francisco, CA, USA) and optimized by Miami Center for AIDS Research at the University of Miami Miller School of Medicine. One primer pair, for *KLF2*, was not validated and results for this gene were not used for further analysis. Amongst the 95 remaining genes were 5 housekeeping genes, 9 epiblast potential specific markers, 12 markers of trophoblast in one or more species, 9 hypoblast markers, 16 chemokine signaling pathway genes, 9 Hippo signaling pathway genes, 13 epigenetic modification gene markers, 14 tight junctions, cell polarity and axon guidance, and another 9 genes of interest.

RNA was extracted using the Qiagen RNeasy Micro Kit (Qiagen) following the manufacturer’s instructions. The RNA isolation procedure included DNase treatment. Elution of RNA was performed in a volume of 20 μL (7 + 7 μL). The High Capacity cDNA Reverse Transcription Kit (Applied Biosystems) was used for cDNA synthesis following manufacturer’s instructions. The cDNA was stored at − 20 °C until further use. The Cells Direct Kit (Life Technologies) was used for preamplification to perform 18 cDNA synthesis cycles, followed by exonuclease I treatment and loading into the microfluidic chip (1:2 dilution). The 96.96 dynamic array Fluidigm integrated fluidic circuit was used to perform PCR. The limit of detection was set as a Ct value greater than 27 cycle. The delta Ct (dCt) values were calculated by the difference between the Ct and the geometric mean of the Ct values for the five housekeeping genes (*ACTB, GAPDH*, *H2AFZ, HPRT1* and *SDHA*) was calculated and used to obtain the delta Ct (dCt) values of the other genes. The fold change (2^−dCt^) was analyzed statistically.

### Experiments

For Experiment 1, it was tested whether knockdown of *PARM1* would compromise competence of embryos to develop to the blastocyst stage, alter TE and ICM differentiation and affect transcript abundance in blastocysts. Embryos were produced in vitro as indicated above. The experiment was performed in 4 replicates. For each replicate, embryos were treated at 22 hpi with vehicle (a negative control containing the same volume of diluent used to prepare the GapmeRs), 5 μM scrambled GapmeR (another negative control to account for non-specific effects of GapmeR) or 5 μM anti-*PARM1* GapmeR added to the culture medium. For each replicate, the percent of presumptive zygotes that cleaved was evaluated at day 3.5 after fertilization and percent of presumptive zygotes and cleaved embryos that became blastocysts were evaluated at day 7.5 after fertilization (Fig. 2). Blastocysts were collected at day 7.5 to either measure gene expression (Figs. 1 and 4) or blastocyst cell number (Fig. 3). Gene expression for *PARM1* was performed by quantitative PCR in 4 pools per treatment of 12–20 blastocysts each (Fig. 1). In addition, expression of 96 genes by high-throughput PCR was determined for 4 pools of 12–15 blastocysts each (Fig. 5). A similar number of embryos were pooled for each treatment in each replicate. The total number of blastocysts evaluated for number of ICM and TE cells by immunofluorescence was 62 (produced in 3 replicates).

Experiment 2 was performed to test whether knockdown of *PARM1* would affect transcript abundance in compact morulae. Embryos were produced and treated as described for Experiment 1 except that compact morulae were collected at day 5.5 after fertilization. Gene expression for *PARM1* was performed by quantitative PCR for 4 pools per treatment of 17–20 compact morula each (Fig. 1) and expression of 96 genes was determined thought high-throughput PCR for 4 pools of 12–15 compact morulae each (Fig. 4). A similar number of embryos were pooled for each treatment in each replicate.

Experiment 3 was designed to test whether negative effects of heat shock on development of morulae to the blastocyst stage would be enhanced in embryos in which transcript abundance of *PARM1* was reduced by GapmeR treatment. Embryos were produced in vitro and cultured with GapmeR treatments (vehicle, 5 μM scrambled GapmeR, or 5 μM anti-*PARM1* GapmeR). At day 5.5 after fertilization, embryos from each treatment were subjected to a heat shock of 41 °C for 24 h or were continued to be cultured at 38.5 °C (i.e., control). All embryos were cultured at 38.5 °C thereafter. The endpoint was the percent of cleaved embryos that became blastocysts at day 7.5 after fertilization (Fig. 6). The number of replicates was 4 and there were an average of 310 presumptive zygotes per treatment.

The purpose of Experiment 4 was similar to that of Experiment 3 except that ER stress was induced with the glycosylation inhibitor tunicamycin [19]. Embryos were produced in vitro and cultured with GapmeR treatments (vehicle, 5 μM scrambled GapmeR, or 5 μM anti-*PARM1* GapmeR). Some oocytes were collected locally and some oocytes were purchased from a commercial in vitro fertilization company (Vytelle, Hermiston, OR, USA). At day 5.5 after fertilization, embryos from each treatment group received treatment for tunicamycin by replacement of 2.5 μL of medium in the culture drop with 2.5 μL SOF-BE2 containing the diluent vehicle for tunicamycin (control) or tunicamycin (0.5 μg/mL tunicamicyn so that the final concentration would be 0.05 μg/mL) (Fig. 7). The number of replicates was 3 and there were an average of 200 presumptive zygotes per treatment.

Two experiments were performed to test whether knockdown of *PARM1* would affect gene expression of three genes involved in ER stress response in compact-morula (Experiment 5) and blastocysts (Experiment 6) (Fig. 8). Embryos were produced in vitro and cultured with GapmeR treatments (vehicle, 5 μM scrambled GapmeR, or 5 μM anti-*PARM1* GapmeR) as described above. Four pools of 15–20 compact morulae collected at day 5.5 after fertilization and 4 pools of 10–15 blastocysts collected at Day 7.5 after fertilization were used to measure mRNA for *ATF6, DDIT3* and *EIF2AK3* by PCR.

### Statistical analysis

The generalized linear mixed models procedure (Proc GLIMMIX) of SAS (SAS Institute Inc., Cary, NC, USA) was used to evaluate effects of treatment on the percent of presumptive zygotes that cleaved and the percent of presumptive zygotes or cleaved embryos that developed to the blastocyst stage. Each embryo was considered as an individual observation. Cleavage and development to the blastocyst stage were analyzed as binary variables (0 = did not occur; 1 = occurred). Treatments (GapmeR, temperature and tunicamicyn) were considered as fixed effects and replicate was considered a random effect. Orthogonal contrasts were used to partition treatment variance into individual comparisons as follows: Anti-*PARM1* GapmeR vs controls (scrambled GapmeR and vehicle) and scrambled GapmeR vs vehicle.

Treatment effects on other variables (cell number and gene expression calculated as fold-change relative to housekeeping genes) were determined by analysis of variance using the generalized linear models procedure (Proc GLM) of SAS. The model for cell number included the effect of treatment as a fixed effect. The model for gene expression included effect of treatment and replicate as fixed effects. Orthogonal contrasts were used as described above. In all cases, *P* < 0.05 was considered significant and *P* < 0.10 a statistical tendency.

## Additional file

Additional file 1: Effects of GapmeR treatment on expression of 90 genes at the compact morula (**Table S1**) and blastocyst (**Table S2**) stages. Data are least-squares means + SEM of fold-change expression relative to the geometric means of five housekeeping (*ACTB, GADPH, HPRT1, H2AF2*, and *SDHA*). Highlighted genes represent those where there was a significant effect of treatment. (XLSX 19 kb)

## Data Availability

The datasets used and/or analysed during the current study are available from the corresponding author on reasonable request..

## Abbreviations

COC: Cumulus-oocyte complex
DPBS: Dulbecco’s phosphate-buffered saline
ER: Endoplasmic reticulum
HEPES-TALP: HEPES -Tyrode’s albumin lactate pyruvate (IVF-TALP)
ICM: Inner cell mass
IVF-TALP: In-vitro fertilization-Tyrode’s albumin lactate pyruvate
PVP: Polyvinylpyrrolidone
SOF-BE2: Synthetic oviduct fluid – bovine embryo 2
TE: Trophectoderm
